# Supplementary material for: Function and regulation of plant ARGONAUTE proteins in response to environmental challenges: a review
Source: PeerJ. 2024 Mar 26;12:e17115. doi: 10.7717/peerj.17115 (PMC10979746; doi:10.7717/peerj.17115)
Supplement: Supplemental Information 1 [file peerj-12-17115-s001.docx]

| **AGO18** | **Gene ID** |
| --- | --- |
| AGO18a | Sspon.02G0007830-1A |
| AGO18b | Sspon.01G0024300-1A |
| AGO18c | Sspon.01G0024300-3D |
| AGO18d | Sspon.02G0007830-2D |

AGO18a: Sspon.02G0007830-1A is non-translating CDS, so its protein seq is not available

>Sspon.01G0024300-1A:Sspon.01G0024300-1A-mRNA-1 peptide: Sspon.01G0024300-1A-mRNA-1:cds pep:protein_coding

MARHRRGRGRRGGRQANPPHTELGGRFPAYDDRDSLFTAGALPFHTKEFEVTLSAGGDKKMDRKYKVVINHAATISLLQLRMLLAGYPTDIPAQALLVLDTVLRDVVSNERDDMKCAAIATKDRTLGVDAWKGLYQSIRSTQNCLSLIADVSSSVFVQPLLLIEFVQRILKMDVVDRNLTKPEYDKLLKVLRGVRIEVTHLGDNRRRKHRIVGLSANPTNDLSFQSSGATTTVINYFREIYILDLKYKSLPCIIARSEQNPIYLPIEVCKIVPRQCYQKKLEASQVSTLRKSACIHPEPEQSCHQIVDQEKYKRTKRANEFDIEVDDNLTAVDARVLLPPNLKYHDSGSQKTGFPMNGYWNMKDKKVINGAKISNWACLNFCEDLSKKDIKEFCFKLAEMSRITGVEFANLKLPIFTARPDQVEDGIHKCYQEAKNKLKDQKIDLLLAILPDKNGSLYGNIKRICETGIGLVSQCCRRSNVFTENSQILSNIAIKINAKAGGRNSVFDDVQKSLPVVSNKPTIIFGAHVSHPSKKKDGSAAPSIASVVASQDWHEVSKYNSVVRAQGHTEEISGLEDIVKELLHAFANESKKKLQQLIFYRVGISEGRFKQVLEKEIPAIEKAWNALYDNEKPQITFIVVQKRHRLRLSPTDNKYKLRSATKKIVEPGTVVDSEICHPAEFDFFLCSQADVKGPRRPVKYLVLRDDNNFTADELQALTNNLCYTYTSGTRSVSIAPPAFYAQKLAHRALVYLAKASDTASASSSGSAGAAAPGGGPKQLPEIKNELKGSMFYC

AGO18c: Sspon.01G0024300-3D is non-translating CDS, so its protein seq is not available

>Sspon.02G0007830-2D-mRNA-1 peptide: Sspon.02G0007830-2D-mRNA-1:cds pep:protein_coding

MASHQRGGGRVGGRGQANPNVAQGQGGRGYGGRGGQYYGGDDNGGRGGGRGGGRGGGRGFDGRGGGYQEGGRGGRGGGGFQEVRGGGRGGGGGYQGGARGGGGGYYEGHGGGRGGGGGGYQGGGRGGRGFQGQGGSDYGRERGLGGLQPPRPDLRQAGPPLADRYAADAAALREKFKTMDIHRDAPMFPARPGFGAVGTPCVVKANHFFVGLVDKGLHHYDVTISPETTLKGIYRQVMSKLVSENRQTELGGRLPAYDGQKSLFTAGELPFKSKEFVVTLPGRVEKRYKVVIKHATAVSLHQLFMLMAGYPTDIPTQALQVLDIVLRDIVLNERNSMEYVAVGRSFFSPLIAPGPKNLGLGVEGWKGFYQSIRPTQKGLSVIVATKSIVHEMDLALLNLQLKKALRGVRIEVTHRGDARRNFESSAGVQKSVADYFREAYKLEMHYDFLPCLQVGSDQRPNYLPMEVCKIVAGQQYRKKLDSQQVSKLMDSTCQRPSDREKNIRQVVEQNDYNRTERASEFGMEVDYRPTSVQARVLPAPTLKYRGTGSESLCCPKDGQWNMIKKQVVHGARVGNWACVNFCHNLPRDVVGKFCSDLVKWSRTTGVDMDNLRLPVYSVRPEQVETDLHKIYQNAQNRLRVQKIDLLLAILPDKNGNLYGNFKRICETEIGIMSQCCLDKNVQSAGPPYFANVAIKINAKFGGRNLEFANPKESLPVVSIEPTIIFGADVTHPAALDDTAPSIASVVASQDWPNVANYNGIARAQGHRKELIDGLEDIVKELLLAFQERSKQRPKQLIFYRDGVSEGQFKQVLEQEIPEIEKAWKALYNEKPKITFIVVQKRHHTRLFPNDRQWTDRSGNILPGTVVDKNICHPTEFDFFLCSHAGIKGTSRPTHYHVLRDDNKFTADALQSLTYNLCY

Sequences source: <https://plants.ensembl.org/Saccharum_spontaneum/Info/Index>
