# Supplementary material for: Function and regulation of plant ARGONAUTE proteins in response to environmental challenges: a review
Source: PeerJ. 2024 Mar 26;12:e17115. doi: 10.7717/peerj.17115 (PMC10979746; doi:10.7717/peerj.17115)
Supplement: Supplementary file 2 [file peerj-12-17115-s002.docx]

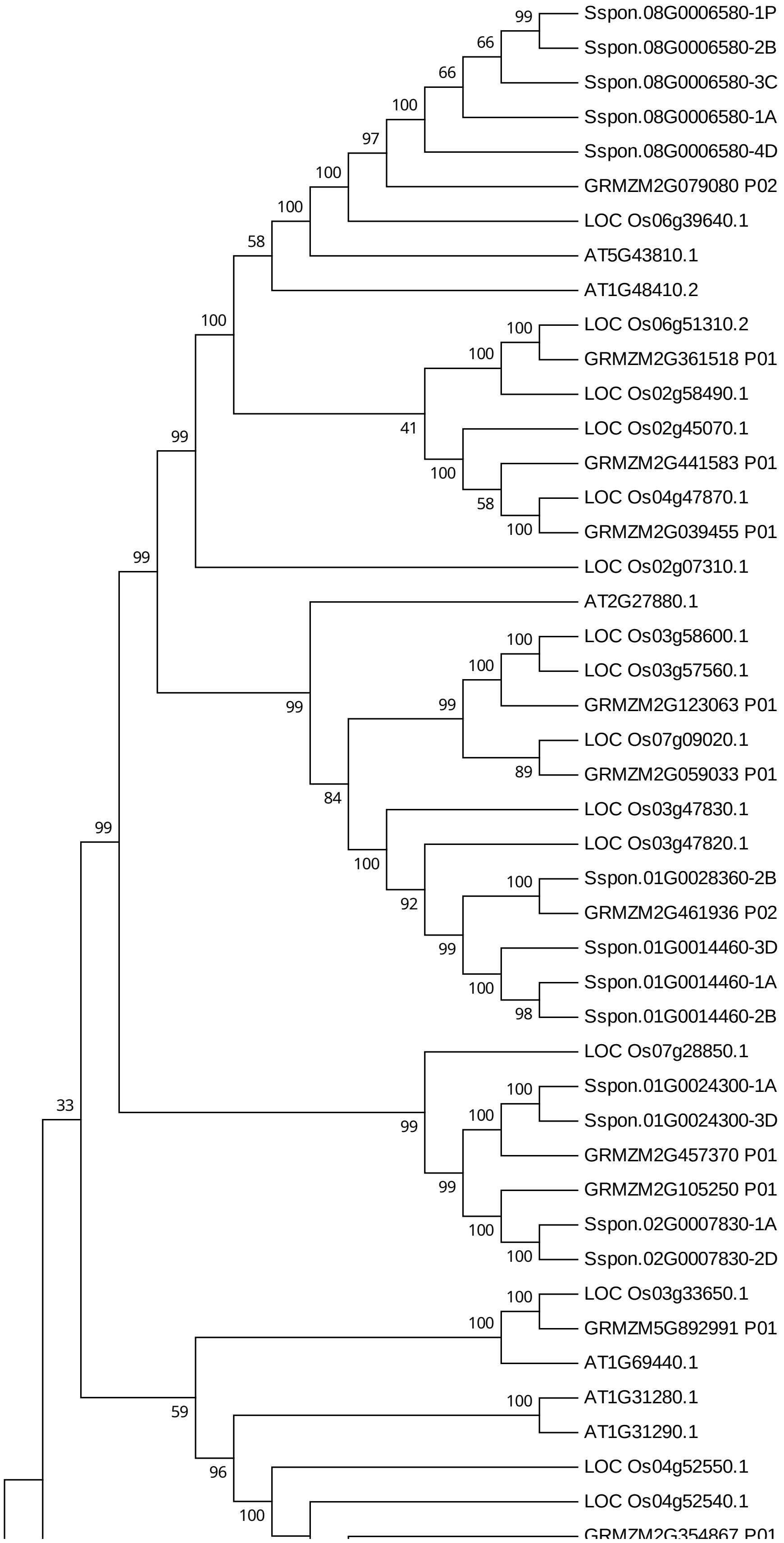
Sspon.08G0006580-1P
Sspon.08G0006580-2B
Sspon.08G0006580-3C
Sspon.08G0006580-1A
Sspon.08G0006580-4D
GRMZM2G079080 P02
LOC Os06g39640.1
AT5G43810.1
AT1G48410.2
LOC Os06g51310.2
GRMZM2G361518 P01
LOC Os02g58490.1
LOC Os02g45070.1
GRMZM2G441583 P01
LOC Os04g47870.1
GRMZM2G039455 P01
LOC Os02g07310.1
AT2G27880.1
LOC Os03g58600.1
LOC Os03g57560.1
GRMZM2G123063 P01
LOC Os07g09020.1
GRMZM2G059033 P01
LOC Os03g47830.1
LOC Os03g47820.1
Sspon.01G0028360-2B
GRMZM2G461936 P02
Sspon.01G0014460-3D
Sspon.01G0014460-1A
Sspon.01G0014460-2B
LOC Os07g28850.1
Sspon.01G0024300-1A
Sspon.01G0024300-3D
GRMZM2G457370 P01
GRMZM2G105250 P01
Sspon.02G0007830-1A
Sspon.02G0007830-2D
LOC Os03g33650.1
GRMZM5G892991 P01
AT1G69440.1
AT1G31280.1
AT1G31290.1
LOC Os04g52550.1
LOC Os04g52540.1
99
66
66
100
97
100
100
58
100
100
100
41
100
58
100
99
99
100
100
99
99
89
84
100
100
92
99
100
98
99
33
100
100
99
99
100
100
100
100
100
59
100
96
100
